# Follow‐up of an occult tuberculosis scar cancer after resection of metastatic lesions

**DOI:** 10.1111/1759-7714.13531

**Published:** 2020-06-22

**Authors:** Mengyao Sun, Yinghui Xu, Xu Wang, Chao Sun, Ye Guo, Guoguang Shao, Zhiguang Yang, Yunpeng Liu, Peng Zhang, Shi Qiu, Kewei Ma

**Affiliations:** ^1^ Cancer Center The First Hospital of Jilin University Changchun China; ^2^ Thoracic Surgery Department The First Hospital of Jilin University Changchun China

**Keywords:** *EGFR* mutation, lung cancer of unknown primary (CUP), metastatic lymph nodes, tuberculosis scar cancer

## Abstract

A 61‐year‐old Chinese man with a history of tuberculosis was found to have a large mass in the left lower lobe and multiple ground‐glass nodules (GGNs) on lung computed tomography (CT). Post‐operative pathology showed lung squamous carcinoma in the left lower lobe and mediastinal lymph node metastases, which were confirmed as lung adenocarcinoma. Multiple gene sequencing was performed, and no relationship was observed between the two primary sites. Chemotherapy consisting of four cycles of gemcitabine plus cisplatin were prescribed for this patient after the operation. After a period of two‐year follow‐up, the lung adenocarcinoma was found to have progressed with new metastases in the right cervical lymph nodes which had the same pathology and gene mutation as the metastatic mediastinal lymph nodes removed two years previously. Meanwhile, a primary lesion was found following PET‐CT scan, and the tuberculosis scar was determined as its point of origin. In conclusion, we have found that a tuberculosis scar is a risk factor of lung cancer, especially adenocarcinoma, and more attention should be paid to close monitoring and follow‐up by clinicians.

## Introduction

Lung cancer is a leading cause of cancer death and represents a major public health problem. Many factors have been reported to be associated with the formation of lung cancer, including smoking, gene mutation, and inflammation.^1^, ^2^ In addition, it has been proposed that a diagnosis of tuberculosis may subsequently increase the risk of lung cancer. The coexistence of tuberculosis and lung cancer is estimated to account for approximately 2%.^3^ Although the two rarely occur together, the possible linkage between tuberculosis and lung cancer development has been studied for several decades. It has been proposed that a tuberculosis scar is associated with an increased risk of lung cancer, as the scarring of the lung after tuberculosis might predispose an individual to lung cancer.^4^, ^5^, ^6^ A tumor which arises from a tuberculosis lesion is known as tuberculosis scar cancer. In this report, we describe a case of lung cancer which originated from a tuberculosis scar which had been found at long‐term follow‐up after resection of the metastatic lesions.

## Case report

**Figure 1:**
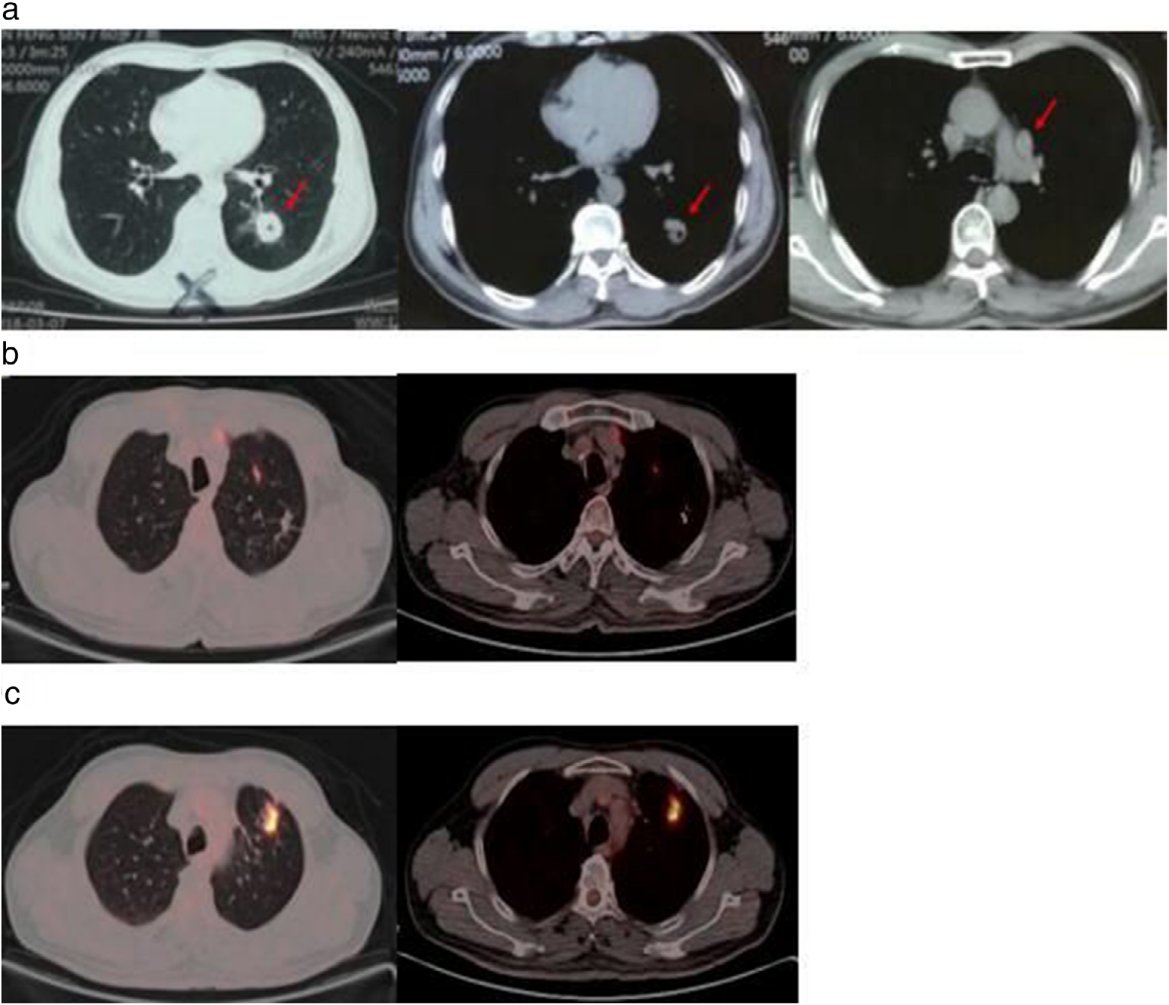
(**a**) Preoperative chest computed tomography (CT) scan indicated a large mass in the left lower lobe and swollen fifth mediastinal lymph node group on 7 March 2018. (**b**) Preoperative chest CT showed tuberculosis in the left upper lobe on 7 March 2018. (**c**) Chest CT showed progressive disease on 5 August 2019.

A large mass in the left lower lobe and multiple ground‐glass nodules (GGNs) were detected by chest computed tomography (CT) scan in a 61‐year‐old Chinese man. He had a history of smoking 40 packs per year and a medical history of pulmonary tuberculosis for more than 10 years that was cured after systemic treatment. No abnormal findings were detected on physical examination. Positron emission tomography‐computed tomography (PET‐CT) showed a cavity‐like hypermetabolic lesion (2.4 cm × 2.3 cm × 2.0 cm in diameter) in the left lower lobe, and the fifth group of mediastinal lymph nodes was enlarged (Fig 1a), suggesting peripheral lung cancer with lymph node metastasis. In addition, multiple GGNs were found in both lungs with undetermined characteristics. Tuberculosis in the upper lobes of both lungs was identified (Fig 1b, Fig S1a). No other distant metastases were detected based on other imaging data. A left lower lobe lobectomy and lymphadenectomy was performed in this patient. Postoperative pathology revealed a squamous cell carcinoma with no driver mutations in the left lower lobe. However, adenocarcinoma harbouring an *EGFR* gene exon 18 mutation (G719A/G719C) was confirmed in the mediastinal lymph nodes, indicating considerable genetic differences and a lack of correlation between the two lesions. We considered that the primary lesion of the metastatic mediastinal lymph nodes might be associated with the tuberculosis scar or GGNs, but no further imaging evidence was found. Therefore, the patient was officially diagnosed with left lung lobe lung cancer (squamous type, pT1cN0M0, stage IA) and mediastinal lymph node metastasis (adenocarcinoma type, pTxN2M0) and was treated with four cycles of chemotherapy (gemcitabine and cisplatin). The status of the case was evaluated as stable disease until July 2019; at this time, the right cervical lymph nodes were enlarged, and adenocarcinoma with *EGFR* gene exon 18 mutation (G719A/G719C) was confirmed by lymph node biopsy, with the same pathology and gene mutation as the metastatic mediastinal lymph nodes that had been removed two years previously. PET‐CT showed a high metabolic malignant mass (2.4 cm × 1.2 cm in diameter) originating from a pulmonary tuberculosis scar in the left upper lobe with multiple lymph node metastases (right neck, bilateral subclavian, left hilar, and mediastinum involved) (Fig. 1c, Fig. S1b). No other metastases were observed. Afatinib was subsequently suggested for this patient.

## Discussion

Here, we present a case which initially manifested with a difference in pathological types between the identified lesion of the lung and metastatic lymph nodes, suggesting there was heterogeneity between them. After two‐years of follow‐up, the origin of the metastatic mediastinal lymph nodes surfaced. It was the scaring area caused by tuberculosis that promoted lung cancer development. Lung cancer may develop in a scar. Lung scar cancer is characterized by a peripheral location and is more likely to be found in the upper lobes. The majority of scar cancers are adenocarcinoma with tumor sizes less than 3 cm in diameter and are found predominantly in the male population.^7^, ^8^, ^9^, ^10^, ^11^ These characteristics are consistent with that of the patient in our report. Lung scar cancer is rare, with a frequency of 7% in all lung cancer patients.^7^ However, a study in South Africa revealed that at least one in five lung cancer patients had radiological evidence of scarring, which might be related to South Africa having one of the highest incidences of tuberculosis.^8^, ^12^ A large‐scale study involving more than 40 000 Chinese patients showed increased lung cancer incidence in patients with tuberculosis.^13^ It has been well documented that tuberculosis played an important role in the formation of lung scar cancer and patients with a history of tuberculosis had shown a two‐fold increase in risk for the subsequent development of lung cancer.^14^, ^15^ It is because the inflammation caused by tuberculosis infection induces the activation of various cytokines, such as tumor necrosis factor (TNF), IL‐1, IL‐6 and many more as well as reactive oxygen species (ROS), which can bind to DNA, leading to genomic alterations. There is a high concentration of collagen III, collagen IV and myofibroblasts, which are characteristics of fibrosis in scars.^16^ Additionally, IL‐3, IL‐4, and TNF‐α, which are produced in high amounts in inflammation, play a key role in the formation of fibrosis.^17^ Chronic inflammation produces and fibrosis causes DNA damage, eventually leading to the activation of oncogenes. It is reported that pulmonary tuberculosis preceded lung cancer in median time of five years (range 2 to 25 years).^6^ In addition, both pulmonary tuberculosis and lung cancer have similar pulmonary manifestations that could mask lung cancers, such as cavitary lesions, miliary pattern, and pleural effusion.^18^, ^19^, ^20^, ^21^ Therefore, the diagnosis of lung cancer can be delayed in patients with a history of pulmonary tuberculosis. The recommendation is that newly diagnosed tuberculosis cases should be followed‐up periodically with chest X‐ray, bronchoscopy, and sputum cytology to screen for the early diagnosis of lung cancer.

The prognosis of lung scar cancer remains controversial. Both Bennett *et al*. and Hukill and Stern found that patients with scar cancers seemed to have a favorable prognosis (five of six patients and three of seven patients surviving five years, respectively).^22^ However, Freant *et al*. reported that the five‐year survival rate of lung scar cancer with surgery was only 5% at the time of resection.^8^ Such poor prognosis of lung scar cancer is due to early lymph node and vascular invasion. As in our case, metastatic lymph nodes were indentified two years prior to the primary lesion. A possible explanation illustrated by Carroll is that the scarring process blocks lymphatic drainage, and carcinogens accumulate within the scar, leading to more extensive vascular and lymphatic seeding.^23^ Freant *et al*. found that lymph node involvement was greater in the scar cancer group, which support the views of Bennett *et al*. and Hukill and Stern that vascular invasion seems to be of little prognostic value and lymph node metastasis is the most important prognostic feature in scar cancer. Early lymph node metastasis is likely to be unique for scar cancer. Therefore, clinicians should be highly alert to the possibility of scar cancer and close monitoring of the lesion condition is essential in patients with lymph node metastasis, especially for patients with a history of tuberculosis.

Factors affecting the prognosis of scar cancer are varied. Previous studies have reported that patients with lung adenocarcinoma who had scar cancer or a history of tuberculosis lesions had a higher probability of having *EGFR* mutations (56%–77%) and the prognosis of patients who had *EGFR* mutations was more favorable than those without mutations (one‐year survival rate, 84.6% vs. 35.4%).^24^, ^25^ However, compared to patients harboring an *EGFR* mutation without a history of tuberculosis, both the progression‐free survival (9.1 months vs. 11.6 months) and the overall survival (19.4 months vs. 24.5 months) after first‐line EGFR‐TKIs were reported to be significantly shorter in the patients with tuberculosis‐related lung adenocarcinoma.^25^ The patient in this case had lymph node metastases harboring a rare *EGFR* mutation (G719A/C) and afatinib had also been administered. and the prognosis should also be followed‐up.

In conclusion, patients with pulmonary tuberculosis scar have an increased risk of lung cancer. For these patients, more attention should be paid to close monitoring and follow‐up. Furthermore, more tuberculosis scar cancer samples are needed to enable further understanding of the biological behavior and potential new treatment.

## Disclosure

The authors declare that they have no conflict of interests.

## Supporting information


**Supplementary Figure S1** (**a**) Sequential preoperative chest CTs shows tuberculosis in the left upper lobe. (**b**) Sequential chest CTs of malignant mass that originated from tuberculosis scar in the left upper lobe.
