# Supplementary figures and images for: Follow‐up of an occult tuberculosis scar cancer after resection of metastatic lesions
Source: Thorac Cancer. 2020 Jun 22;11(8):2347–50. doi: 10.1111/1759-7714.13531 (PMC7396367; doi:10.1111/1759-7714.13531)

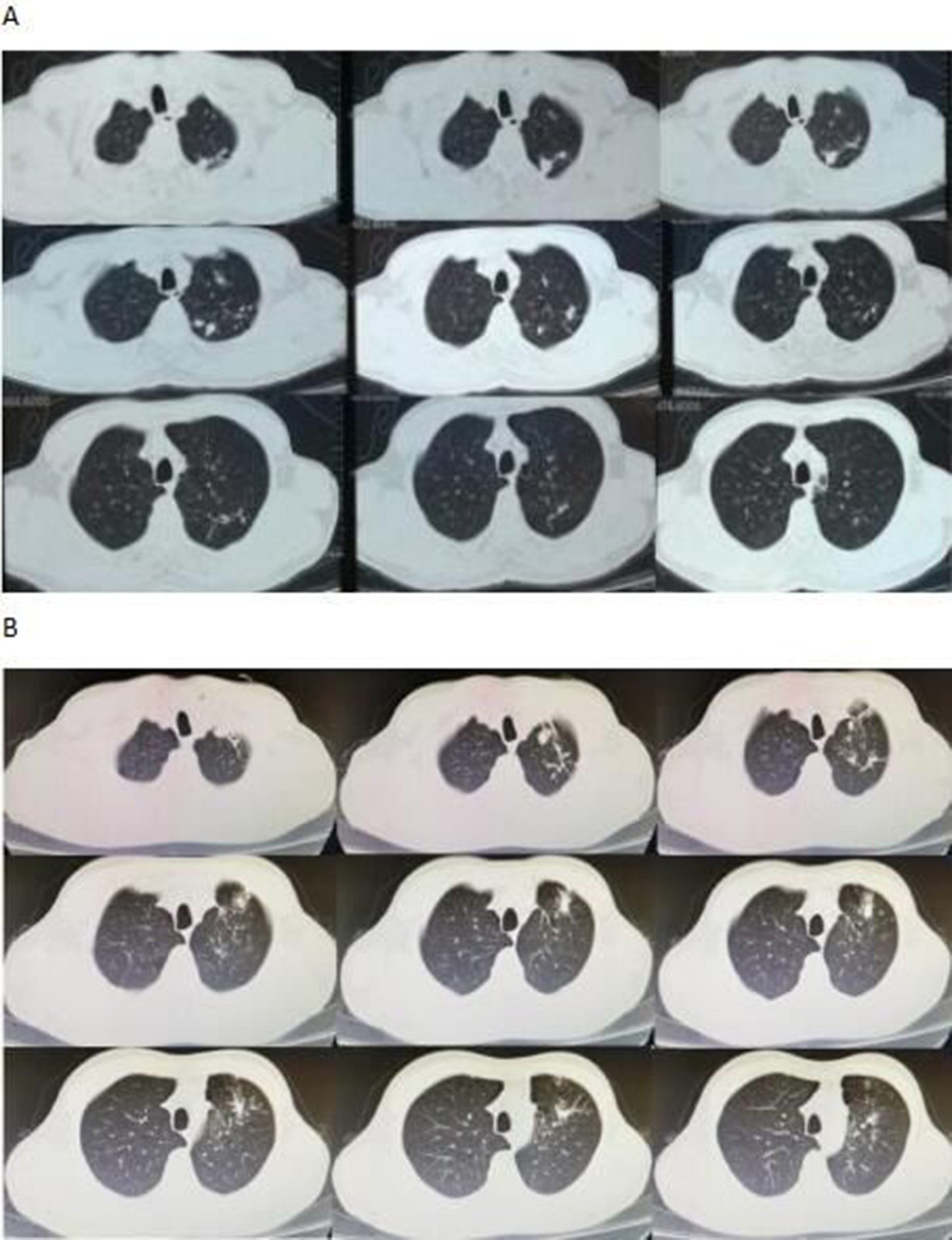

Supplement: Supplementary file 1 — Supplementary Figure S1 (a) Sequential preoperative chest CTs shows tuberculosis in the left upper lobe. (b) Sequential chest CTs of malignant mass that originated from tuberculosis scar in the left upper lobe. [file TCA-11-2347-s001.jpg]
